# Association of risk factors with smoking during pregnancy among women of childbearing age: an epidemiological field study in Turkey: Associação de fatores de risco com tabagismo durante a gravidez em mulheres em idade fértil: estudo epidemiológico de campo na Turquia

**DOI:** 10.1590/1516-3180.2016.021921102016

**Published:** 2017-04-03

**Authors:** Naim Nur

**Affiliations:** I MD. Professor, Department of Public Health, School of Medicine, Cumhuriyet University, Sivas, Turkey.

**Keywords:** Smoking, Prevalence, Women, Pregnancy, Gestational age

## Abstract

**CONTEXT AND OBJECTIVE:**

Smoking during pregnancy is an important risk factor for maternal and infant health that is preventable. This study aimed to investigate the risk factors associated with smoking behavior during pregnancy.

**DESIGN AND SETTING:**

A household-based probability sample survey of 1,510 women was conducted in the center of the city of Sivas, Turkey, between September 2013 and May 2014.

**METHODS:**

The prevalence of smoking during pregnancy was estimated according to independent variables by means of regression analysis.

**RESULTS:**

The prevalence of smoking during pregnancy was 16.5%. Logistic regression showed that being at a relatively young age (odds ratio, OR = 1.92, P = 0.025 for 15-24 age group; and OR = 2.45, P = 0.001 for 25-34 age group), having a low educational level (OR = 1.76, P = 0.032), being unmarried (OR = 1.48, P = 0.002) and living in an extended family (OR = 1.98, P = 0.009) were the factors associated with the risk of smoking during pregnancy.

**CONCLUSIONS:**

Systematic attention should be paid to socioeconomic inequalities, to support women towards quitting smoking before or at an early stage of their pregnancies. Younger women and particularly those in lower socioeconomic groups should be targeted. This will lead to better pregnancy status, especially among young women.

## RESUMO

**CONTEXTO:**

Fumar durante a gravidez é um fator de risco importante para a saúde materna e infantil e que pode ser evitado. O estudo teve como objetivo investigar os fatores de risco associados com o comportamento de fumar durante a gravidez.

**TIPO DE ESTUDO E LOCAL:**

Inquérito por amostragem probabilística de base domiciliar de 1.510 mulheres foi realizado no centro da cidade de Sivas, Turquia, entre setembro de 2013 e maio de 2014.

**MÉTODOS:**

A prevalência do hábito de fumar durante a gravidez foi estimada de acordo com as variáveis independentes por meio da análise de regressão.

**RESULTADOS:**

A prevalência do hábito de fumar durante a gravidez foi de 16,5%. A regressão logística mostrou que estar em idade relativamente jovem *(odds ratio*, OR = 1,92, P = 0,025 por grupo de idade 15-24e OR = 2,45, P = 0,001 para a faixa etária 25-34), com alto nível de escolaridade (OR = 1,76, P = 0,032), sendo solteira (OR = 1,48, P = 0,002) e vivendo em família grande (OR = 1,98, P = 0,009) foram os fatores associados ao risco de fumar durante a gravidez. Parto prematuro e baixo peso ao nascer foram mais frequentes entre as fumantes.

**CONCLUSÕES:**

Atenção sistemática deve ser dada às desigualdades socioeconômicas para apoiar mulheres a parar de fumar antes ou na fase inicial da gravidez, tendo como alvo mulheres mais jovens, e particularmente aquelas de grupos socioeconômicos mais desfavorecidos. Isso conduzirá a um melhor estado de gravidez em mulheres, especialmente às jovens.

## INTRODUCTION

Maternal smoking during pregnancy has been identified as the most preventable source of newborn morbidity and mortality throughout the world. The health problems, economic burden and dependence caused by smoking are predictably greater in vulnerable populations such as pregnant women. Smoking during pregnancy increases the risk of adverse pregnancy outcomes such as perinatal mortality or miscarriage, premature births, low birthweight and small fetuses.^1^^,^^2^^,^^3^^,^^4^

According to a recent study on trends within the prevalence of smoking in developing and developed countries, the prevalence of smoking has decreased significantly worldwide.^5^ However, as suggested by some studies, because the rates of smoking cessation are lower in socioeconomically vulnerable populations, the differences in smoking rates between socioeconomic levels may have increased.^6^^,^^7^

Several variables relating to sociodemographic conditions are closely connected with the likelihood of smoking behavior. Furthermore, smoking behavior is inversely associated with socioeconomic position, such that vulnerable people in the community are more likely to continue their smoking behavior.^8^^,^^9^ Despite considerable public understanding of the dangers of smoking during pregnancy, more than a third of women who smoke continue their smoking behavior during pregnancy.^10^^,^^11^^,^^12^

Research has documented factors relating to smoking behavior during pregnancy and their effect on adverse pregnancy outcomes. The social structure of smoking behavior during pregnancy is largely determined by social factors.^13^^,^^14^^,^^15^ The consequences of pregnancy in situations of low socioeconomic level are determined through internal factors and the habit of cigarette smoking is among these factors.^16^ Up to half of the adverse consequences of pregnancy are caused by smoking behavior among women living under low socioeconomic conditions.^17^ It is even more important to take into account the effect of smoking during pregnancy among women living in different socioeconomic levels in developing countries like Turkey, which have high inequality in maternal and infant mortality rates.

Because of the direct risks to infant health, much attention has been paid to maternal smoking during pregnancy over recent years. However, few data on the characteristics of high-risk populations regarding smoking behavior are available. Previous studies have been limited by several factors. Most of these studies have been conducted in the hospital setting rather than community setting or have had small sample sizes.^11^^,^^12^

## OBJECTIVE

The aim of the present study was to investigate the risk factors associated with smoking during pregnancy.

## METHODS

### Setting and ethics

This cross-sectional population-based study was conducted in the center of the city of Sivas, Turkey, between September 2013 and May 2014. Sivas is a Middle Anatolian city with approximately 625,000 inhabitants. The number of women of reproductive age (15-49 years) in the urban area of this city is about 85,000. Compared with other cities in Turkey, it has an average structure with regard to socioeconomic and demographic conditions.

Women were interviewed in person at their homes. All participants were informed that all information obtained through the interview would be kept confidential. The study was approved by the Ethics Committee of Cumhuriyet University.

### Sampling design

A large sample consisting of 1,510 individuals was used. This sample size was enough to estimate an expected prevalence of smoking during pregnancy of 20% from a large population with a margin of error for a 95% confidence interval of ± 2%.^11^^,^^12^ The target population of the study comprised around 38,000 households in 63 districts. A multistage cluster sampling scheme was used in this study. The sampling scheme was prepared by listing the number of households in each district. The electricity company’s records were used to find the number of households. Firstly, a total of 11 districts were randomly selected. Secondly, the street and street number of the dwelling on the street were selected randomly in each of the districts. Households were sampled with probability proportional to size. Buildings primarily providing short-term or temporary accommodation such as hotels, rental homes, etc., were excluded. Over the study period, to increase the chance of obtaining an interview, two revisits to each household were made on different days of the week. If a household contained more than one eligible woman, one of them was randomly chosen by drawing lots for the interview. The eligible women enrolled into the study were those who had been pregnant at some time during the previous three years (before the time of the interview), who did not present any communication difficulties and who gave their informed consent to participate.

Before starting the face-to-face interview, written and verbal consent was sought from every participant. All interviews were conducted in a quiet room in each respondent’s home, by trained final-year medical students.

### Survey instrument

The survey questionnaire for this study was developed based on the existing literature and was reviewed by two research experts. In order to judge the time needed to administer the questionnaire and to test it for clarity and logical flow, it was piloted with 20 women.

The questionnaire consisted of two sections. The first section of the questionnaire requested demographic information on the participants, including maternal age, current marital status, health insurance, family type (nuclear or extended, i.e. a family that included not only parents and children but also other relatives such as grandparents, aunts or uncles), number of previous deliveries, education level and employment status. Employment status was categorized as an office job, manual work, or unemployed. In this study, subjects who were students or housewives were registered as unemployed. Also in this section, the subjects were asked about the place where they had spent the majority of their lives, whether they had any type of health insurance and about their perceived health status and income level. Based on self-reported data, the annual household income was categorized into two groups: 1 (≤ US$ 7,000) and 2 (> US$ 7,000). Likewise, health-related problems were coded as 1 = present or 0 = absent; the women were asked to self-report whether during their last pregnancy they had had one or more of the following chronic medical conditions: diabetes, hypertension, arthritis, thyroid disorders, migraines, asthma, gastrointestinal disorders, cancer or physical disability.

In the second section of the questionnaire, the women were asked whether they had smoked during their last pregnancy. Smoking was defined as cigarette smoking at least once a week. Furthermore, alcohol consumption was determined through the frequency of drinking, defined as: often (least once a week), occasionally (rarely, less than one beverage per month) or never.

### Independent and dependent variables

Associations of the following independent contextual variables were considered to be relevant to smoking during pregnancy: age, maternal education, marital status, employment status, family type and annual household income.

### Statistical analysis

The Statistical Package for the Social Sciences (SPSS Inc., Chicago, IL, USA) for Windows, version 16.0, was used for the data analysis. Categorical data were expressed as percentages. Quantitative data were presented as mean ± standard deviation, SD. To evaluate associations between dependent and independent variables, bivariate analysis using the chi-square test was performed. Also, multiple logistic regression analyses were performed to assess which variables were significantly associated with smoking behavior as dependent variables. Thus, age, maternal education, marital status, employment status, family type and annual household income were included in the model for smoking behavior as independent variables. Purposeful selection of candidate variables was done based on a bivariate P-value < 0.15. The fit of the multiple logistic models was assessed using the Akaike information criterion (AIC). The model with the lowest AIC was accepted as the best-fitting model. To determine multicollinearity among the variables, collinearity diagnostic tests were conducted. In none of the cases were the “tolerance values” less than 0.2, and no variance inflation factor was greater than 10; P-values less than 5% were considered to be statistically significant.

## RESULTS

Out of the total of 1,510 women initially in the sample, 24 did not meet the inclusion criteria. A further 187 excluded themselves due to domestic commitments, while 26 could not be contacted at their home. Consequently, the survey involved 1,273 eligible women who agreed to participate in a face-to-face interview, thus yielding a survey response rate of 84.3%. In the present survey, the retrospective evaluation took place approximately 1.3 years (1.3 ± 1.1) after pregnancy.

### Characteristics of the sample

All the study participants were from urban areas. Their mean age was 36.4 ± 7.9 years (range: 15-49 years). Most of the participants (62%) were over 34 years old. As presented in Table 1, with regard to the demographic characteristics of the sample over the year prior to the survey, more than half of the participants (57.2%) were living in nuclear families, and approximately 80% of them did not have a university degree. Most of the participants (85.5%) had simple health insurance, 91% were married, more than 25% were employed and 81% had an annual household income of ≤ US$ 7,000 (Table 1).

**Table 1:**
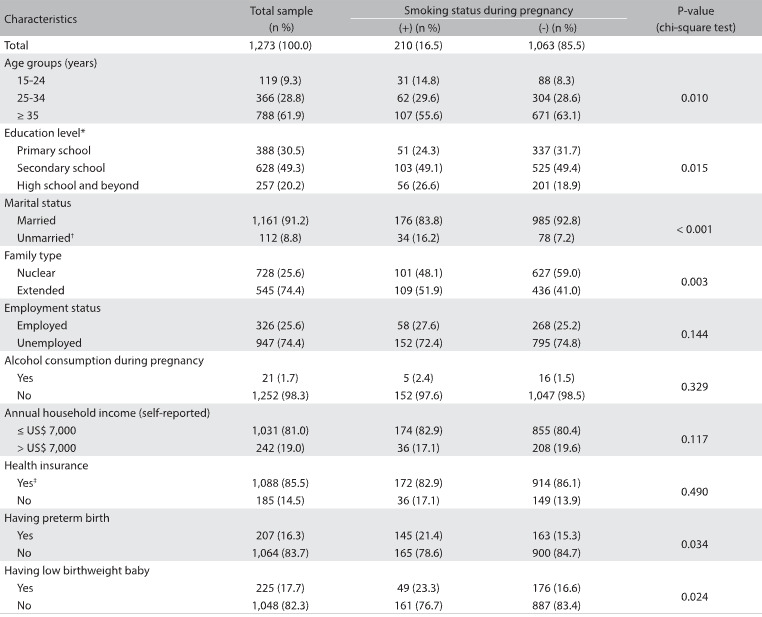
Characteristics of the participants: total sample and sample according to smoking status during pregnancy

^*^The normal age ranges for starting to attend the different school levels in Turkey are 6-7 years old for primary school (mandatory), 10-11 years old for secondary school and 13-14 years old for high school. ^†^Including single, separated, divorced and widowed women. ^‡^Including the Greencard that is given to poor individuals by the Turkish Health Ministry for free healthcare but which is limited to emergency care.

### Prevalence and factors relating to smoking during pregnancy

The prevalence of self-reported smoking during pregnancy was found to be 16.5% in this study sample (Table 1). Bivariate analysis results comparing smokers and nonsmokers are shown in Table 1. Regarding smoking behavior, there were differences among the women in terms of age, education, marital status and type of family. However, there were no significant differences regarding their health insurance, employment status, alcohol consumption or annual household income.

Bivariate analysis showed that, compared with nonsmokers, smokers were more likely to be under 25 years of age (14.8% versus 8.3%; P = 0.010) and had a higher level of education (26.6% versus 18.9%;P = 0.014). More smokers were unmarried (16.2% versus 7.2%; P = 0.003) and were living in extended families (51.9% versus 41.0%; P = 0.003), compared with nonsmokers. Table 2 presents variables associated with being a smoker during pregnancy, based on multivariate logistic regression analysis. Being at a relatively young age (odds ratio, OR = 1.92, r = 0.65, P = 0.025 for the 15-24 age group; and OR = 2.45, r = 0.90, P = 0.001 for the 25-34 age group), having primary educational level (OR = 1.76, r = 0.56, P = 0.032), being unmarried (OR = 1.48, r = 0.48, P = 0.002) and living in an extended family (OR = 1.98, r = 0.69, P = 0.009) were significantly associated with a risk of smoking during pregnancy (Table 2).

**Table 2:**
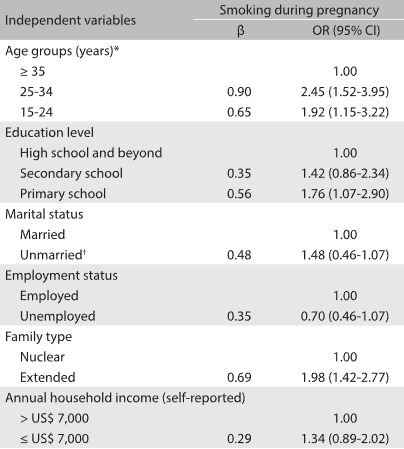
Relationship between sociodemographic variables and smoking during pregnancy, based on multivariate logistic regression analysis, with odds ratios and 95% confidence intervals (n = 1,273)

All statistically significant P-values are in bold type; ^*^during pregnancy; ^†^including single, separated, divorced and widowed women; OR = odds ratio; CI = confidence interval.

## DISCUSSION

This survey highlights the social factors involved in smoking during pregnancy among women living in a Middle Anatolian city. It has been estimated that 14%-30% of pregnant women continue smoking during pregnancy, including 17% in England and Wales,^18^ 15% in Romania and in Australia,^19^^,^^20^ 14% in the United States,^21^ 13% in Israel^22^ and 30% in Poland.^23^ In line with these recent studies, the prevalence of smoking in the present study was 16.5%. However, the prevalence of smoking found in the present study was lower than in other studies on Turkish women, including 18% among 499 pregnant women^11^ or 23% among 256 women.^12^ These disparities in prevalence levels may be due to differences between geographic locations or due to hospital setting designs.^19^ Furthermore, it should be noted here that the true prevalence of smoking during pregnancy may be difficult to discern, because of possible underreporting in studies that depend on self-reporting.^24^ Also, sociocultural norms discouraging smoking may lead women to fail to disclose their true smoking status during pregnancy.

The harmful behavior of smoking contributes substantially towards deterioration of maternal and child health, especially among those living with socioeconomic disadvantages. Previous studies have reported that women who continued to smoke throughout the pregnancy had about twice the risk of adverse pregnancy outcomes, compared with those who did not smoke or reduced their smoking habit.^11^^,^^18^

The smoking pattern demonstrated in this study confirms previous research findings that smoking is more prevalent among relatively young women and among women who live in poor socioeconomic circumstances.^11^^,^^12^^,^^15^^,^^22^ This may be explained by the marketing campaigns that are designed specifically to target young women.^25^

In this study, it was found that women for whom primary school was their highest education level had around twice as much risk of smoking during pregnancy. This was in line with previous research that showed that there were prominent socioeconomic differences between women who continued smoking during pregnancy and those who did not.^26^^,^^27^

An association between marital status and smoking during pregnancy was found in the present study. There was around a 1.5 times greater risk of continuing smoking during pregnancy among unmarried women, and this finding was consistent with previous research indicating that unmarried women had the highest prevalence of smoking.^26^^,^^27^ A similar association was also found in the present study between women who were living in an extended family and continuing to smoke during pregnancy. This may be explained by the fact that in addition to women’s roles as mothers and homemakers, their participation in the workforce has increased in recent years. From this perspective, smoking not only is a coping mechanism for escaping from or avoiding negative emotions, but also is important in relation to strong feelings of autonomy for finding one’s own place in society.^28^^,^^29^

In this survey, the retrospective evaluation took place around 1.3 years after pregnancy. Previous research has been called into question with regard to recall bias, because bias in recollecting data and behavior is a severe risk in using retrospective methods. Nonetheless, concerning smoking during pregnancy, a previous study demonstrated that recollections relating to pregnancy were still accurate five or six years afterwards.^30^ Pregnancy is an important event within life and the social stigma relating to smoking during pregnancy is assumed to provide a model for this accuracy. Hence, in the present survey, the time of 1.3 years after pregnancy that elapsed should not be considered problematic with regard to validity. Because of the characteristics of cross-sectional designs, self-reporting may be an important limitation in this survey. However, evidence from population-based studies has demonstrated that self-reporting of smoking status has a high level of validity.^31^^,^^32^ In the present study, a face-to-face design was used rather than a self-administered design, and the fact that the information was obtained from the women in their homes means that the data regarding sociodemographic and socioeconomic determinants is likely to have been more reliable and more accurate.

### Limitations

There were some limitations to this study. Firstly, the study was undertaken on a community-based sample in a single city. Because of the nature of cross-sectional designs, the findings from the study sample can possibly be generalized to the population of women of childbearing age, but no assessment of temporal relationships and thus potentially causal relationships between the variables was possible. Secondly, all the data were dependent on the women’s perceptions and their accuracy of recall. No objective measurements were used to validate the participants’ responses regarding smoking behavior. Furthermore, underreporting bias is common in relation to socially undesirable behavior like smoking. Finally, because smoking during pregnancy is considered to be an unacceptable and avoidable exposure, there was a possibility of reporting bias.

## CONCLUSION

Smoking rates during pregnancy and adverse outcomes from pregnancy were found to vary according to social circumstances. Systematic attention should be paid to socioeconomic inequalities, to support women towards quitting smoking before or at an early stage of their pregnancies. Younger women and particularly those in lower socioeconomic groups should be targeted. This will lead to better pregnancy status, especially among young women.

## References

[1] Ashford KB, Hahn E, Hall L (2010). The effects of prenatal secondhand smoke exposure on preterm birth and neonatal outcomes. J Obstet Gynecol Neonatal Nurs.

[2] Bailey BA, Byrom AR (2007). Factors predicting birth weight in a low-risk sample: the role of modifiable pregnancy health behaviors. Matern Child Health J.

[3] Burns L, Mattick RP, Wallace C (2008). Smoking patterns and outcomes in a population of pregnant women with other substance use disorders. Nicotine Tob Res.

[4] McCowan LM, Dekker GA, Chan E (2009). Spontaneous preterm birth and small for gestational age infants in women who stop smoking early in pregnancy: prospective cohort study. BMJ.

[5] Ng M, Freeman MK, Fleming TD (2014). Smoking prevalence and cigarette consumption in 187 countries, 1980-2012. JAMA.

[6] Schaap MM, Kunst AE, Leinsalu M (2008). Effect of nationwide tobacco control policies on smoking cessation in high and low educated groups in 18 European countries. Tob Control.

[7] Bosdriesz JR, Willemsen MC, Stronks K, Kunst AE (2015). Socioeconomic inequalities in smoking cessation in 11 European countries from 1987 to 2012. J Epidemiol Community Health.

[8] Siahpush M, Borland R (2001). Socio-demographic variations in smoking status among Australians aged &gt; or = 18 multivariate results from the 1995 National Health Survey. Aust N Z J Public Health.

[9] Greenhalgh EM, Bayly M, Winstanley MH, Scollo MM, Winstanley MH (2015). Trends in the prevalence of smoking by socio-economic status. Tobacco in Australia: Facts and issues.

[10] Ortendahl M, Näsman P (2007). Perception of smoking-related health consequences among pregnant and non-pregnant women. Am J Addict.

[11] Uncu Y, Ozcakir A, Ecran I, Bilgel N, Uncu G (2005). Pregnant women quit smoking; what about fathers? Survey study in Bursa Region, Turkey. Croat Med J.

[12] Ergin I, Hassoy H, Tanik FA, Aslan G (2010). Maternal age, education level and migration: socioeconomic determinants for smoking during pregnancy in a field study from Turkey. BMC Public Health.

[13] Cnattingius S (2004). The epidemiology of smoking during pregnancy: smoking prevalence, maternal characteristics, and pregnancy outcomes. Nicotine Tob Res.

[14] Goy J, Dodds L, Rosenberg MW, King WD (2008). Health-risk behaviours: examining social disparities in the occurrence of stillbirth. Paediatr Perinat Epidemiol.

[15] Villalbí JR, Salvador J, Cano-Serral G, Rodríguez-Sanz MC, Borrell C (2007). Maternal smoking, social class and outcomes of pregnancy. Paediatr Perinat Epidemiol.

[16] Gissler M, Meriläinen J, Vuori E, Hemminki E (2003). Register based monitoring shows decreasing socioeconomic differences in Finnish perinatal health. J Epidemiol Community Health.

[17] Kramer MS, Séguin L, Lydon J, Goulet L (2000). Socio-economic disparities in pregnancy outcome: why do the poor fare so poorly?. Paediatr Perinat Epidemiol.

[18] National Statistics. The Information Centre (2006). Statistics on smoking: England 2006.

[19] Seybold DJ, Broce M, Siegel E, Findley J, Calhoun BC (2012). Smoking in pregnancy in West Virginia: does cessation/reduction improve perinatal outcomes?. Matern Child Health J.

[20] Meghea CI, Rus D, Rus IA, Summers Holtrop J, Roman L (2012). Smoking during pregnancy and associated risk factors in a sample of Romanian women. Eur J Public Health.

[21] Tong VT, Jones JR, Dietz PM (2009). Trends in smoking before, during, and after pregnancy - Pregnancy Risk Assessment Monitoring System (PRAMS), United States, 31 sites, 2000-2005. MMWR Surveill Summ.

[22] Polanska K, Hanke W, Sobala W (2005). [Characteristic of the smoking habit among pregnant women on the base of the test "Why am I a smoker?"]. Przegl Lek.

[23] Fisher N, Amitai Y, Haringman M (2005). The prevalence of smoking among pregnant and postpartum women in Israel: a national survey and review. Health Policy.

[24] Dietz PM, Homa D, England LJ (2011). Estimates of nondisclosure of cigarette smoking among pregnant and nonpregnant women of reproductive age in the United States. Am J Epidemiol.

[25] World Health Organization (2008). WHO Report on the Global Tobacco Epidemic, 2008. The MPOWER Package.

[26] Schneider S, Schütz J (2008). Who smokes during pregnancy? A systematic literature review of population-based surveys conducted in developed countries between 1997 and 2006. Eur J Contracept Reprod Health Care.

[27] Ebert LM, Fahy K (2007). Why do women continue to smoke in pregnancy?. Women Birth.

[28] Rigbi A, Yakir A, Sarner-Kanyas K, Pollak Y, Lerer B (2011). Why do young women smoke? VI. A controlled study of nicotine effects on attention: pharmacogenetic interactions. Pharmacogenomics J.

[29] Lumley J, Chamberlain C, Dowswell T (2009). Interventions for promoting smoking cessation during pregnancy. Cochrane Database Syst Rev.

[30] Karim E, Mascie-Taylor CG (1997). The association between birthweight, sociodemographic variables and maternal anthropometry in an urban sample from Dhaka, Bangladesh. Ann Hum Biol.

[31] Raum E, Arabin B, Schlaud M, Walter U, Schwartz FW (2001). The impact of maternal education on intrauterine growth: a comparison of former West and East Germany. Int J Epidemiol.

[32] Hensley Alford SM, Lappin RE, Peterson L, Johnson CC (2009). Pregnancy associated smoking behavior and six year postpartum recall. Matern Child Health J.

